# The Risk of Hospitalization and Mortality After Breakthrough SARS-CoV-2 Infection by Vaccine Type: Observational Study of Medical Claims Data

**DOI:** 10.2196/38898

**Published:** 2022-11-08

**Authors:** Meghana Kshirsagar, Md Nasir, Sumit Mukherjee, Nicholas Becker, Rahul Dodhia, William B Weeks, Juan Lavista Ferres, Barbra Richardson

**Affiliations:** 1 Microsoft Redmond, WA United States; 2 Insitro Inc South San Francisco, CA United States; 3 Paul G Allen School of Computer Science & Engineering University of Washington Seattle, WA United States; 4 Department of Biostatistics and Global Health University of Washington Seattle, WA United States

**Keywords:** breakthroughs, vaccines, Pfizer, Moderna, Janssen, SARS-CoV-2, COVID-19, coronavirus, infectious disease, viral infection, vaccination, breakthrough infection, public health, health policy, decision making, booster vaccine, mortality, hospitalization, healthcare system

## Abstract

**Background:**

Several risk factors have been identified for severe COVID-19 disease by the scientific community. In this paper, we focus on understanding the risks for severe COVID-19 infections after vaccination (ie, in breakthrough SARS-CoV-2 infections). Studying these risks by vaccine type, age, sex, comorbidities, and any prior SARS-CoV-2 infection is important to policy makers planning further vaccination efforts.

**Objective:**

We performed a comparative study of the risks of hospitalization (n=1140) and mortality (n=159) in a SARS-CoV-2 positive cohort of 19,815 patients who were all fully vaccinated with the Pfizer, Moderna, or Janssen vaccines.

**Methods:**

We performed Cox regression analysis to calculate the risk factors for developing a severe breakthrough SARS-CoV-2 infection in the study cohort by controlling for vaccine type, age, sex, comorbidities, and a prior SARS-CoV-2 infection.

**Results:**

We found lower hazard ratios for those receiving the Moderna vaccine (*P*<.001) and Pfizer vaccine (*P*<.001), with the lowest hazard rates being for Moderna, as compared to those who received the Janssen vaccine, independent of age, sex, comorbidities, vaccine type, and prior SARS-CoV-2 infection. Further, individuals who had a SARS-CoV-2 infection prior to vaccination had some increased protection over and above the protection already provided by the vaccines, from hospitalization (*P*=.001) and death (*P*=.04), independent of age, sex, comorbidities, and vaccine type. We found that the top statistically significant risk factors for severe breakthrough SARS-CoV-2 infections were age of >50, male gender, moderate and severe renal failure, severe liver disease, leukemia, chronic lung disease, coagulopathy, and alcohol abuse.

**Conclusions:**

Among individuals who were fully vaccinated, the risk of severe breakthrough SARS-CoV-2 infection was lower for recipients of the Moderna or Pfizer vaccines and higher for recipients of the Janssen vaccine. These results from our analysis at a population level will be helpful to public health policy makers. Our result on the influence of a previous SARS-CoV-2 infection necessitates further research into the impact of multiple exposures on the risk of developing severe COVID-19.

## Introduction

Despite widespread COVID-19 vaccination, high community levels of SARS-CoV-2 circulating throughout the United States have led to many breakthrough SARS-CoV-2 infections [1-3]. Breakthrough infections, where fully vaccinated individuals who are exposed to SARS-CoV-2 get infected, are generally uncommon (0.02% of fully vaccinated individuals reported developing breakthrough infections in a Washington state cohort [4]) and are generally less severe than infections in unvaccinated individuals [5,6]. There now exists a large body of literature studying the risk factors for severe COVID-19 disease, much of which has involved studies in unvaccinated populations [7-10], prior to the large-scale availability of vaccines. Studies on how these risks vary after vaccination are fewer in comparison, mostly focused on vaccine effectiveness in preventing SARS-CoV-2 infections or the influence of specific variants on vaccine effectiveness [11].

The impact of underlying factors on breakthrough infections are quite challenging to understand outside of randomized, placebo-controlled, double-blind field trials due to variation in their severity, distribution in the population, and contribution to transmission [11,12]. Early studies have found that a third dose of vaccine reduces the viral load in breakthrough infections, even for newer variants such as delta and omicron [13,14]. To understand the comparative advantages of the various vaccines [15], it is important to know the rate of severe COVID-19 disease leading to hospitalization or death among individuals who are fully vaccinated [16], as this will help policy makers.

While the risk of breakthrough SARS-CoV-2 infection has recently been reported by type of vaccine [17], little information exists regarding the risk of *hospitalization* or *mortality* by vaccine type for breakthrough infections [1]. In addition, while a prior SARS-CoV-2 infection is associated with a lower risk of breakthrough infection, it is unknown how large an effect a prior infection has on the severity of breakthrough COVID-19 infections, should one occur [18]. There has been a growing need for retrospective studies on severe breakthrough infections to address the misinformation and vaccine hesitancy in social and public spheres [19].

In this paper, we used de-identified US medical claims records from Change Healthcare to estimate the risk of hospitalization and death, by vaccine type, age, sex, comorbidity factors and previous SARS-CoV2 infection, among SARS-CoV-2 breakthrough infections that occurred between March 10, 2021, and October 14, 2021.

## Methods

### Ethics Approval and Consent to Participate

This study does not constitute as human subjects research due to the use and reporting of only deidentified observational data as determined by the human subjects committee of the University of Washington and thus does not require the review and approval by the institutional review board at the University of Washington.

### Data Source

Our study uses de-identified US medical claims records from Change Healthcare collected over a period from March 1, 2020, to October 14, 2021, encompassing over 100 million records from over 8 million patients. Medical claims data contain details about a patient’s interaction with the medical system, which are needed for the accurate billing of the transactions. Each claims record contains patient demographic information, International Classification of Diseases, 10th Revision (ICD-10) codes indicating primary diagnosis and secondary diagnoses, place of diagnosis, ICD-10 codes of procedures performed, patient status at the end of the visit, dates pertaining to the event (where different “from” and “to” dates indicate longer visits whereas the same “from” and “to” dates are for outpatient visits).

Our claims data set includes primarily open claims and a subset of closed payer claims that are normalized for analytics purposes. The open claims are derived from broad-based health care sources and consist of all medical claims that Change Healthcare processes and for which it has the usage rights. The closed claims are derived directly from the payer (ie, health insurance provider) and capture nearly all events that occur during the patient’s enrollment period. Roughly 95% of the claims used for this study are commercial, and 5% are Medicare Advantage or other types of plans.

### Study Population

Our data set of 8.18 million individuals contains only COVID-19 positive patients, defined as patients with at least one claims record with the ICD-10 diagnosis codes of “U07.1” or “U07.2” in any diagnosis field. We limited our analysis to individuals who had a primary diagnosis of “U07.1” (this is indicated by the principal diagnosis code, which encodes the primary diagnosis rendered by the medical facility or the primary cause of the visit). This ICD-10 diagnosis code indicates a COVID-19 diagnosis where the virus was identified in a lab-confirmed report. We exclude patients for whom the code of U07.1 appears in the “other diagnosis” fields, which contain the list of diagnoses made in addition to the primary cause of visit, which can be any other medical condition such as cancer. We also exclude patients with the code U07.2, which indicates a non-lab–confirmed COVID-19 diagnosis.

Subsequently, fully vaccinated individuals are identified by looking for procedure codes encoding the second doses of Pfizer (0002A) and Moderna (0012A) vaccines and the first dose of the Janssen (0031A) vaccine. We do not exclude patients with missing first dose claims records (~5% of the final study cohort), because patients who went to vaccination camps and were not required to provide insurance information would have missing first dose claims records. Some of these ~5% patients with missing first dose claims records may have had mixed vaccines (eg, Pfizer for the first dose and Moderna for the second dose). Since we did not believe that this will be a significant fraction of the vaccinated population, we do not exclude them. Breakthrough patients were defined as those who had a primary, lab-confirmed COVID-19 diagnosis at least 14 days after the date of vaccination. Please see Figure S1 in Multimedia Appendix 1 for a flow diagram showing the criteria used for cohort selection.

### Hospitalization and Mortality

We explicitly identify hospitalization by looking for claims where the claim type is “institutional” or “professional” and the bill type indicates an “inpatient” facility. We also look at the dates associated with the hospital stay and only consider patients whose admission duration was at least 2 days (derived from the “admission_from” and “admission_to” date fields). For mortality, we look at the patient status code and consider all codes indicating “expired.” As described already, we only consider cases where the primary diagnosis was COVID-19 for both hospitalization and expiration. The patient status code is available for all hospitalized patients but only for 42% of the outpatients (who went to clinics). Among patients who had the patient status code available, we found only 17 (0.22%) deaths out of a total of 7843 outpatients; therefore, we consider outpatients with missing patient status to be alive.

### Study Period

COVID-19 vaccinations began in the United States in late December 2020. By late February 2021, the Pfizer-BioNTech (Pfizer), Moderna, and Johnson & Johnson (J&J/Janssen) vaccines were all approved for emergency use authorization. The Pfizer and Moderna vaccination drives started much earlier, in late December (Figures S2 and S3 in Multimedia Appendix 1), as compared to that of the Janssen vaccines, which also saw a stall in vaccine rates in mid-April (Figure S4 in Multimedia Appendix 1). To keep the *COVID-19 exposure* of the individuals taking any of the 3 vaccines consistent, we use the same study window, though we have data for Pfizer and Moderna from late December. We construct our cohort to consist of individuals who were fully vaccinated between March 10, 2021, and April 27, 2021, the period during which all 3 vaccines were being widely administered. Every individual in this cohort was followed from the date of vaccination of each individual up to the end of the study period, October 14, 2021. The study period over the entire cohort is thus March 10, 2021, to October 14, 2021. In Figure S6 in Multimedia Appendix 1, we show statistics showing the number of days of follow-up after full vaccination by vaccine type in our study cohort.

### Comorbidities

Preexisting comorbidities were defined based on ICD-10 codes assigned to medical encounters, which contain pointers to previously diagnosed conditions, using claims records during the 6-month period from March 2020 to September 2020. This period does not overlap with the study period, so events during the study period will not also be counted as comorbidities. The Elixhauser comorbidity index [20] was used to define comorbid conditions. This index has a series of codes that define comorbidities with each code mapping to one or several ICD-10 diagnosis codes. For example, the Elixhauser code “BLDLOSS” (blood loss) includes the following four ICD-10 diagnosis codes: D50.0, O90.81, O99.02, and O99.03. We provide the index that we used and the corresponding ICD-10 codes in Multimedia Appendix 2. We also show the relative abundance of comorbidities in our cohort, by vaccine type, in Table S2 and Figure S5 in Multimedia Appendix 1.

### Previous COVID-19 Infection

Since some of the individuals in our cohort may have had a COVID-19 infection during the year 2020, we introduce an additional feature to encode the effect of already being infected with COVID-19. This feature is “yes” if we see a claim involving a COVID-19 diagnosis in any diagnosis field, in the period from March 1, 2020, to the beginning of the study period, March 10, 2021.

### Statistical Methods

Date of full vaccination was defined as 14 days after (1) a single Janssen vaccine, (2) the second Moderna vaccine dose, or (3) the second Pfizer vaccine dose. Cox proportional hazards regression was used to estimate univariate hazard ratios (HRs) and multivariable HRs in a model including the following features: age (categorized), sex (male and female), vaccine type, Elixhauser comorbidities (encoded as independent binary variables), and SARS-CoV-2 infection prior to the first dose of vaccination (yes or no). We remove the negligible number of individuals with sex=unknown. We also model interactions between vaccine type and all other covariates as well as previous infection and all other covariates but find that none were statistically significant. Further, the interaction terms had a negligible impact on the hazard ratios of the other terms and were thus removed for greater clarity in the results. All analyses were performed using the “coxph” function from the R package “survival” (R Foundation for Statistical Computing) [21].

## Results

Our study includes 19,815 fully vaccinated patients with breakthrough SARS-CoV-2 infections between March 10, 2021, and October 14, 2021. Of those patients, 11,339 (57.22%) received the Pfizer vaccine, 5480 (27.66%) received the Moderna vaccine, and 2996 (15.12%) received the Janssen vaccine. Breakthrough cases receiving Janssen were younger than those receiving Pfizer or Moderna and had a slightly greater proportion of male patients (Table 1). Breakthrough cases receiving Moderna had a greater proportion of patients with COVID-19 prior to vaccination.

Risk of hospitalization and mortality among breakthrough cases increased with older age and was higher for male patients (Table 2). In multivariable analyses controlling for age, male sex, comorbidities, and prior SARS-CoV-2 infection, the risk of hospitalization was the lowest for breakthrough cases receiving the Moderna vaccine (adjusted hazard ratio [aHR]: 0.42, 95% CI 0.35-0.5; *P*<.001), comparably low for Pfizer vaccinated individuals (aHR: 0.45, 95% CI 0.39-0.53; *P*<.001), compared with that for the recipients of the Janssen vaccine. The comorbidities with statistically significant HRs for hospitalization or mortality from a breakthrough SARS-CoV-2 infection include severe liver disease, moderate and severe renal failures, alcohol abuse, chronic lung disease, coagulopathy, cancers, anemia, seizures, and arthritis (Table S1 in Multimedia Appendix 1).

**Table 1:** Characteristics of SARS-CoV-2 breakthrough infections cohort tracked from March 10, 2021, to October 14, 2021. Prevalence of comorbidities by vaccine type is shown in Figure S5 in Multimedia Appendix 1.

Variable			Pfizer (n=11,339), n (%)		Moderna (n=5480), n (%)		Janssen (n=2996), n (%)		Overall (n=19,815), n (%)
**Age range (years)**									
	0-20	108 (0.95)		31 (0.57)		34 (1.13)		173 (0.87)	
	20-35	1005 (8.86)		455 (8.30)		337 (11.25)		1797 (9.07)	
	35-50	1801 (15.88)		795 (14.51)		722 (24.10)		3318 (16.74)	
	50-64	3663 (32.30)		1684 (30.73)		1224 (40.85)		6571 (33.16)	
	64-80	4007 (35.34)		2041 (37.24)		580 (19.36)		6628 (33.45)	
	>80	755 (6.66)		474 (8.65)		99 (3.30)		1328 (6.70)	
Sex (male)			5032 (44.4)		2360 (43.06)		1385 (46.2)		8777 (44.23)
SARS-CoV2 infection before vaccination			1536 (13.5)		1137 (20.7)		437 (13.9)		3090 (15.6)

**Table 2:** Correlates of hospitalization and mortality after breakthrough SARS-CoV-2 infection, estimated from Cox proportional hazards models. We show the adjusted hazard ratio (aHR) and the 95% CI for the significant correlates (*P* values indicated via superscripts d, e, and f). An aHR of <1.0 indicates a lower risk of hospitalization or mortality as compared to the baseline population for that covariate (analogously, aHR>1.0 indicates a higher risk than the baseline). Hazard ratios (HRs) of comorbidities are shown in Table S1 in Multimedia Appendix 1.

Variable			Hospitalization (n/pys^a^)		Hospitalization, univariate HR (95% CI)^b^		Hospitalization, multivariate aHR (95% CI)^b^		Mortality (n/pys)		Mortality, univariate HR (95% CI)^c^		Mortality, multivariate aHR (95% CI)^c^
**Vaccine**													
	Pfizer	20.1		0.55 (1.8-0.46)^d^		0.45 (0.39-0.53)^d^		2.6		0.68 (1.5-0.45)		0.43 (0.28-0.65)^d^	
	Moderna	19.2		0.59 (1.7-0.5)^d^		0.42 (0.35-0.5)^d^		2.3		0.61 (1.6-0.37;^e^ (*P*=.04)		0.38 (0.23-0.62)^d^	
	Janssen	26.5		1.0		1.0		3.0		1.0		1.0	
**Age range (years)**													
	0-20	1.9		0.29 (0.04-2.1)		0.30 (0.04-2.2)		1.9		7.48 (0.78-71.9)		7.8 (0.81-75)	
	20-35	1.9		0.27 (0.15-0.5)^d^		0.29 (0.15-0.54)^d^		0.0		0 (0)		0 (0)	
	35-50	6.8		1.0		1.0		0.3		1.0		1.0	
	50-64	16.9		2.08 (1.62-2.7)^d^		2.1 (1.6-2.7)^d^		1.8		5.82 (1.8-19.0)^f^ (*P*=.004)		5.98 (1.8-20)^f^ (*P*=.003)	
	64-80	31.7		2.96 (2.33-3.7)^d^		3.32 (2.6-4.2)^d^		3.9		12.30 (3.9-38.9)^d^		14.20 (4.5-45)^d^	
	>80	52.9		4.35 (3.34-5.7)^d^		4.99 (3.8-6.5)^d^		9.1		24.60 (7.6-79.7)^d^		29.10 (8.9-95)^d^	
**Sex**													
	Female	17.5		1.0		1.0		2.2		1.0		1.0	
	Male	25.0		1.38 (1.23-1.5)^d^		1.25 (1.1-1.4)^d^		3.0		1.26 (0.93-1.7)		1.11 (0.82-1.5)	
**SARS-CoV2 infection before vaccination**													
	No	21.9		1.0		1.0		2.7		1.0		1.0	
	Yes	7.5		0.56 (0.4-0.78)^d^		0.57 (0.41-0.80)^f^ (*P*=.001 for above aHR)		0.5		0.21 (0.05-0.84)^f^ (*P*<.01 for above aHR)		0.22 (0.05-0.91)^e^ (*P*=.04 for above aHR)	

^a^Incidence per 100 person years.

^b^n=19,815; events=1140.

^c^n=19,815; events=159.

^d^*P*<.001.

^e^*P*<.05.

^f^*P*<.01.

We see a similar trend with the risk of mortality for breakthrough cases, with the risk being the lowest for those receiving the Moderna vaccines (aHR: 0.38, 95% CI 0.23-0.62; *P*<.001) and comparably lower for Pfizer recipients (aHR: 0.43, 95% CI 0.28-0.65; *P*<.001) as compared to that for Janssen recipients. Finally, as expected, the protection offered by vaccines was enhanced for breakthrough cases who already had a previous SARS-CoV-2 infection. These individuals were 40% less likely to be hospitalized due to COVID-19 (aHR: 0.57, 95% CI 0.41-0.80; *P*=.001) and four times less likely to die of COVID-19 (aHR: 0.22, 95% CI 0.05-0.91; *P*=.04), when compared to those without a prior SARS-CoV-2 infection independent of age, sex, comorbidities, and vaccine type.

We repeat this analysis by excluding the population who had a prior SARS-CoV-2 infection for completeness and show the resulting HRs Table S3 in Multimedia Appendix 1.

## Discussion

### Principal Findings

Using medical claims data, we found that the risk of hospitalization in SARS-CoV-2 breakthrough infections was lower for those receiving the Moderna and Pfizer vaccines compared to those receiving the Janssen vaccine. The risk of mortality was similarly low in breakthrough infections who received Pfizer and Moderna vaccines compared to those receiving the Janssen vaccine. There was no statistically significant difference between the HRs of Pfizer and Moderna for both risks. We also found older age, male sex, and certain comorbidities to be risk factors for hospitalization and mortality in breakthrough infections. Further, we found that risk of hospitalization was 40% less and risk of death was 75% less in SARS-CoV-2 breakthrough infections among individuals who already had a SARS-CoV-2 infection prior to their vaccination compared with fully vaccinated individuals without a previous SARS-CoV-2 infection. While other studies have reported lower risk of breakthrough infection with previous SARS-CoV-2 infection [18], our study analyzes both hospitalization and mortality and shows that the immunity provided by previous infection seems to increase the protection provided by vaccines, against severe COVID-19, independent of vaccine type, age, comorbidities, and sex. Since our cohort only consists of individuals who were all fully vaccinated, this is by no means a comparison of vaccine-induced immunity against acquired immunity from previous infections.

Excluding patients who had COVID-19 infection prior to vaccination from our Cox regression analysis results in a similar HR for hospitalization risk in patients who received the Pfizer (aHR=0.42) and Moderna (aHR=0.41) vaccines (Table S3 in Multimedia Appendix 1). This might be explained by the fact that 20.7% of patients who received Moderna had a prior COVID-19 infection as compared to ~13% of patients who received Pfizer. Hence, removing all patients with prior COVID-19 infection reduced the influence of the additional immunity that some of Moderna-vaccinated individuals had.

A number of studies have found that age has a direct effect on the risk of severe COVID-19 disease [22,23]. We find that the proportion of the elderly cohort in our data set who were hospitalized is much higher than the proportion of the younger cohort (Figure S7 in multimedia Appendix 1). In addition, we find a higher HR for the elderly subset of our study cohort (aHR=2.1 for age>50, aHR=3.3 for age>65, and aHR=5.0 for age>80, as compared to the baseline age group of 35-50 years).

Our findings comparing vaccine types are similar to those reported by the Centers for Disease Control and Prevention for mortality but provide additional information by vaccine type [1,16]. There have been several studies on individual risk factors such as age [22], specific comorbidities [7], focused populations such as Veterans [24], or large-scale projects such as OpenSAFELY, which involved 17 million unvaccinated patients [10]. Our work advances this body of literature by analyzing vaccine type, age, sex, and 39 different comorbidities in a large cohort of breakthrough patients from the general US population. Some of the risk factors that we find for severe breakthrough SARS-CoV-2 infections, such as age, male gender, and certain comorbidities (eg, chronic lung infection, kidney disease, and cancers) are similar to what has been reported in prior studies of SARS-CoV-2 infections among unvaccinated individuals [8,23,25]. However, we find that some risk factors found by initial studies such as hypertension are not a risk factor for breakthrough COVID-19 hospitalization or death (aHRs of 0.75 and 0.59, respectively), neither are diabetes or congestive heart failure. We instead find that both moderate and severe renal failure are significant risk factors, independent of age or other factors, which agrees with other large-scale studies such as OpenSAFELY [10] and the Global Burden of Disease collaboration [26] which identified that worldwide chronic kidney disease is the most prevalent risk factor for severe COVID-19. Even mild impairment of renal function has been found to be an independent risk factor for COVID-19 infection, hospitalization, and mortality [27].

Lastly, to understand the association between outcomes and the *time of vaccination*, we incorporate a variable indicating the number of days between full vaccination and the onset of the surge in infections caused by the delta variant. However, our population-based data set is inadequate to derive any significant conclusions vis-à-vis the best time for vaccination in anticipation of a surge.

### Limitations

Limitations of our study include, first, a lack of access to data on unvaccinated individuals or those who had a negative SARS-CoV-2 test result. The former is due to the lack of a medical claims record for vaccinations that were done in vaccination drives and camps; the absence of a vaccination-related claim in our data set therefore does not imply an unvaccinated individual. Second, our medical claims source consists of mostly privately insured individuals and can thus miss people who may be susceptible to the most adverse outcomes. Another caveat of our data set is that most of the claims are open claims, which have the benefit of capturing a patient’s activities over a longer time frame regardless of their insurance provider, but do not necessarily track all medical encounters of patients.

### Conclusions

Our findings add to the growing literature regarding the risk factors for severe breakthrough SARS-CoV-2 infections in fully vaccinated individuals, where we identify the influence of age, sex, and comorbidities that are risk factors; importantly, we found that previous SARS-CoV-2 infections can provide additional protection over that offered by vaccines against severe disease. Our results also necessitate further studies on the optimal number of vaccine doses to protect from the most severe breakthrough SARS-CoV-2 infections. An important strength of our study is that we consider US-wide breakthrough hospitalizations covering a broad demographic and compare all 3 vaccines, whereas most previous studies lack specific data on Janssen.

## Appendix

Multimedia Appendix 1 Supplemental data.

Multimedia Appendix 2 Elixhauser comorbidities table.

## Abbreviations

aHR: adjusted hazard ratio
HR: hazard ratio
ICD-10: International Classification of Diseases, 10th Revision
