# Supplementary material for: The Risk of Hospitalization and Mortality After Breakthrough SARS-CoV-2 Infection by Vaccine Type: Observational Study of Medical Claims Data
Source: JMIR Public Health Surveill. 2022 Nov 8;8(11):e38898. doi: 10.2196/38898 (PMC9645422; doi:10.2196/38898)
Supplement: Multimedia Appendix 1 [file publichealth_v8i11e38898_app1.docx]

Supplemental Online Content: Risk of hospitalization and mortality after breakthrough SARS-CoV-2 infection by vaccine type utilizing medical claims data

Authors: Meghana Kshirsagar^1^ (BS, MS, PHD), Md Nasir^1^ (BS, MS, PHD), Sumit Mukherjee^4^ (BS, MS, PHD), Nicholas Becker^1^ (BS, MS), Rahul Dodhia^1^ (BS, PhD), William B Weeks^1^ (MD, PHD, MBA), Juan Lavista Ferres^1^ (BS, MS), Barbra A. Richardson^2,3^ (BS, MS, PhD).

^1^AI for Good Research Lab, Microsoft Corporation, Redmond, USA.

^2^Departments of Biostatistics and Global Health, University of Washington, Seattle, USA.

^3^Vaccine and Infectious Disease Division, Fred Hutch Cancer Research Center, Seattle, USA.

^4^Insitro Labs, USA

Corresponding author: Dr. Meghana Kshirsagar, email: [Meghana.Kshirsagar@microsoft.com](mailto:Meghana.Kshirsagar@microsoft.com)

Postal address: 1 Microsoft Way, Redmond, WA 98052, phone: +1 (425) 4218258.

## **Contents**

Figure S1: Study cohort and vaccine-level statistics of the population

Table S1: Adjusted hazards ratio (aHR) of the 39 Elixhauser comorbidities, for Hospitalization and Mortality After Breakthrough SARS-CoV-2 Infection, estimated from Cox proportional hazards models.

Figure S2: Distribution of vaccination dates in individuals getting Pfizer vaccine’s second dose.

Figure S3: Distribution of vaccination dates in individuals getting Moderna vaccine’s second dose.

Figure S4: Distribution of vaccination dates in individuals getting Janssen vaccine

Table S2: Prevalence of comorbidities by vaccine type. For each vaccine type, the count of patients from that vaccine group that had each comorbidity is shown.

Figure S5: Relative prevalence of comorbidities by vaccine type. For each vaccine type, the percentage (%) of vaccinated patients that had each comorbidity is shown.

Figure S6: Follow-up duration, following full vaccination, for patients by vaccine-type.

Table S3. Correlates of Hospitalization and Mortality After Breakthrough SARS-CoV-2 Infection, after removing the patients with “prior COVID-19 infection” from the study cohort.

Figure S7. Number of patients getting hospitalized per month, split by age-group. A much larger proportion of senior individuals were hospitalized as compared to younger individuals.

Figure S1. Study cohort and vaccine-level statistics of the population. To create “Our cohort of breakthrough patients”, we filter the set of N=40,880 patients to only include those who were fully vaccinated (i.e. got their second dose for Pfizer/Moderna and first dose for Janssen) between the dates of Mar 10^th^, 2021, to Apr 27^th^, 2021 (date range highlighted in red). We had data for these patients between Mar 1^st^, 2020, to Oct 15^th^, 2021.


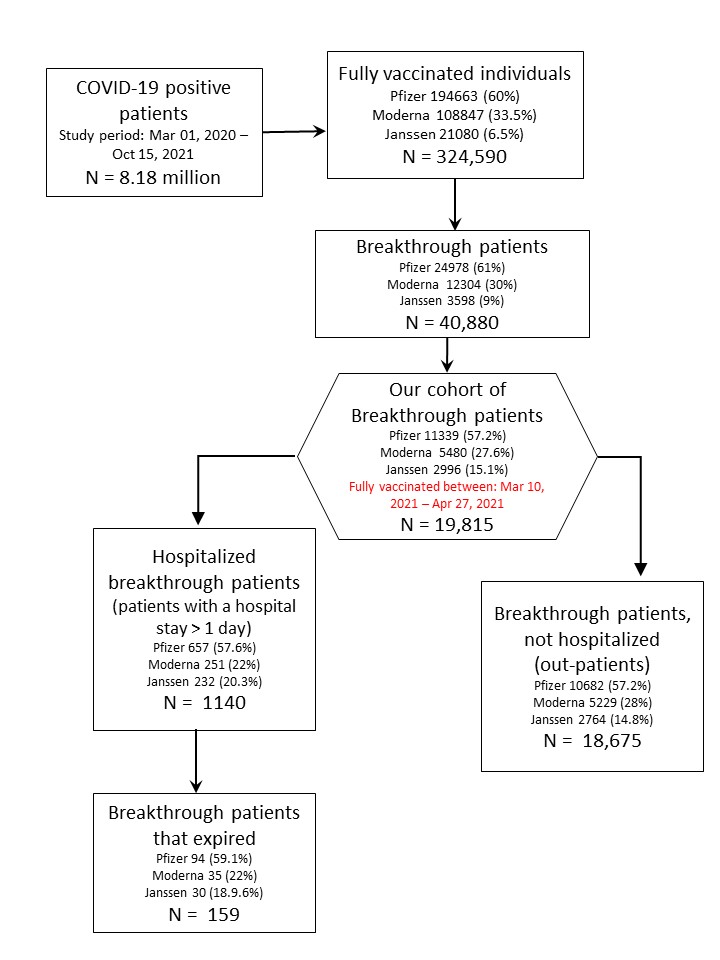


Table S1. Adjusted hazards ratio (aHR) of the 39 Elixhauser comorbidities, for Hospitalization and Mortality After Breakthrough SARS-CoV-2 Infection, estimated from Cox proportional hazards models.

| Elixhauser Comorbidity | Incidence percentage (%) n/population | Hospitalization Multivariate aHR (95% CI) N=19,815 | Mortality Multivariate aHR (95% CI) N=19,815 |
| --- | --- | --- | --- |
| AIDS | 0.79 | 1.20 (0.62--2.34) | 1.35 (0.18--9.94) |
| Alcohol abuse | 2.23 | 1.61 (1.14--2.27)** | 1.90 (0.76--4.74) |
| Deficiency anaemia | 15.83 | 1.25 (1.05--1.49)* | 1.68 (1.04--2.71)* |
| Rheumatoid arthritis/collagen vascular diseases | 6.43 | 1.26 (1.02--1.55)* | 1.14 (0.62--2.09) |
| Blood loss anaemia | 1.82 | 0.85 (0.60--1.21) | 0.78 (0.30--2.05) |
| Congestive Heart Failure | 7.00 | 1.18 (0.97--1.43) | 1.56 (0.94--2.58). |
| Chronic Lung disease | 16.75 | 1.22 (1.05--1.42)** | 0.84 (0.54--1.30) |
| Coagulopathy | 4.37 | 1.35 (1.09--1.66)** | 1.08 (0.63--1.86) |
| Cerebrovascular disease (on admission) | 6.26 | 0.90 (0.73--1.10) | 0.73 (0.42--1.26) |
| Cerebrovascular disease (other) | 1.20 | 1.04 (0.63--1.71) | 0.99 (0.31--3.14) |
| Depression | 14.60 | 1.06 (0.89--1.26) | 0.81 (0.49--1.35) |
| Diabetes Complicated | 14.91 | 1.11 (0.91--1.36) | 0.98 (0.57--1.70) |
| Diabetes Uncomplicated | 19.11 | 1.17 (0.97--1.41). | 1.16 (0.70--1.93) |
| Drug Abuse | 2.48 | 1.00 (0.69--1.45) | 0.30 (0.04--2.20) |
| Hypertension Complicated (HTN_CX) | 11.35 | 1.22 (0.99--1.51). | 1.43 (0.83--2.46) |
| Hypertension Uncomplicated (HTN_UNCX) | 37.98 | 0.75 (0.64--0.88)*** | 0.59 (0.38--0.93)* |
| Hypothyroidism | 11.82 | 1.04 (0.87--1.23) | 1.03 (0.65--1.64) |
| Cancer - Leukemia | 1.08 | 1.71 (1.18--2.45)** | 3.16 (1.41--7.07)** |
| Liver disease (mild) | 8.34 | 0.92 (0.75--1.12) | 0.50 (0.26--0.98)* |
| Liver disease (severe) | 0.74 | 1.86 (1.25--2.79)** | 7.23 (2.99--17.46)*** |
| Cancer -Lymphoma | 1.03 | 1.47 (1.05--2.07)* | 1.16 (0.45--2.99) |
| Metastatic cancer | 1.17 | 1.53 (1.06--2.22)* | 2.10 (0.82--5.35) |
| Dementia | 1.61 | 0.95 (0.67--1.34) | 1.07 (0.44--2.59) |
| Neurological disorders (Movement) | 2.20 | 1.12 (0.83--1.51) | 0.85 (0.35--2.06) |
| Other Neurological disorders | 3.34 | 0.98 (0.74--1.28) | 0.90 (0.44--1.83) |
| Seizures and epilepsy | 2.36 | 1.36 (1.01--1.84)* | 0.77 (0.29--2.06) |
| Obesity | 24.18 | 1.01 (0.87--1.17) | 1.05 (0.69--1.60) |
| Other thyroid disorders | 5.18 | 0.81 (0.61--1.07) | 0.78 (0.35--1.76) |
| Paralysis | 1.40 | 0.89 (0.54--1.45) | 2.01 (0.68--5.99) |
| Peripheral vascular disorders | 10.08 | 1.01 (0.85--1.20) | 1.46 (0.94--2.27). |
| Psychosis | 4.81 | 0.97 (0.72--1.29) | 0.40 (0.12--1.32) |
| Pulmonary circulation disorders | 2.17 | 1.08 (0.82--1.42) | 0.84 (0.41--1.75) |
| Renal failure (moderate) | 7.83 | 1.31 (1.08--1.59)** | 1.05 (0.64--1.72) |
| Renal failure (severe) | 4.97 | 1.69 (1.36--2.11)*** | 2.11 (1.23--3.60)** |
| Cancer without metastasis, malignant | 6.07 | 0.90 (0.73--1.11) | 0.71 (0.40--1.27) |
| Cancer without metastasis, in situ | 1.31 | 1.15 (0.77--1.70) | 3.52 (1.74--7.12)*** |
| Peptic ulcer disease | 1.48 | 1.06 (0.72--1.54) | 0.55 (0.16--1.91) |
| Valvular disease | 7.97 | 1.03 (0.86--1.23) | 1.34 (0.85--2.12) |
| Weight loss | 3.76 | 1.16 (0.92--1.45) | 1.71 (1.00--2.92)* |

*P<0.05

**P<0.01

***P<0.001

**Methods:**

**Data Source:**

Roughly 95% of the claims used for this study are commercial and 5% are Medicare Advantage/other types of plans. Every medical claims record contains information about the diagnoses (in the form of ICD-10 codes), the procedures performed and prescribed drugs. The claims in our dataset include primarily open claims, and a subset of closed payer claims which are normalized for analytics purposes providing sound directional insight for this study. The open claims are derived from broad based healthcare sources and consists of all the medical claims that Change Healthcare processes and for which they have the rights to use. The closed claims are derived from the payer and capture nearly all events that occur during the patient’s enrollment period.

Figure S2. Distribution of vaccination dates in individuals getting Pfizer vaccine’s second dose. At the peak of Pfizer’s vaccination drive, our data has more than 3000 people were getting vaccinated per day.

Figure S3. Distribution of vaccination dates in individuals getting Moderna vaccine’s second dose. While the distribution looks similar to that of Pfizer, the height of the peak is much lower, at around 1750.

Figure S4. Distribution of vaccination dates in individuals getting Janssen vaccine, the earliest date being 24^th^ Feb, 2021. The peak is much lower at around 1000 individuals per day.

Table S2. Prevalence of comorbidities by vaccine type. For each vaccine type, the count of patients from that vaccine group that had each comorbidity is shown.

|  | **Number of patients with the condition** | | |
| --- | --- | --- | --- |
| **Comorbidity** | **Pfizer** | **Moderna** | **Janssen** |
| AIDS | 190 | 122 | 44 |
| Alcohol abuse | 529 | 359 | 117 |
| Deficiency anemias | 3917 | 2448 | 759 |
| Arthropathies | 1748 | 947 | 201 |
| Chronic blood loss anemia | 481 | 266 | 71 |
| Congestive heart failure | 1753 | 1074 | 322 |
| Chronic pulmonary disease | 4500 | 2397 | 640 |
| Coagulopathy | 1032 | 673 | 264 |
| Cerebrovascular disease (on admission) | 1627 | 967 | 225 |
| Cerebrovascular disease (other) | 297 | 186 | 55 |
| Depression | 3903 | 2038 | 632 |
| Diabetes with chronic complications | 3674 | 2290 | 747 |
| Diabetes without chronic complications | 4910 | 2851 | 841 |
| Drug abuse | 516 | 406 | 194 |
| Hypertension, complicated | 2823 | 1751 | 536 |
| Hypertension, uncomplicated | 10095 | 5511 | 1490 |
| Hypothyroidism | 3230 | 1654 | 437 |
| Cancer - Leukemia | 340 | 125 | 21 |
| Liver disease, mild | 2205 | 1209 | 342 |
| Liver disease, moderate to severe | 201 | 106 | 25 |
| Cancer - Lymphoma | 300 | 139 | 23 |
| Cancer - Metastatic | 363 | 139 | 24 |
| Dementia | 411 | 261 | 52 |
| Neurological disorders affecting movement | 582 | 333 | 76 |
| Other neurological disorders | 876 | 469 | 160 |
| Seizures and epilepsy | 601 | 319 | 142 |
| Obesity | 6516 | 3336 | 1033 |
| Other thyroid disorders | 1440 | 744 | 149 |
| Paralysis | 335 | 227 | 67 |
| Peripheral vascular disease | 2601 | 1522 | 414 |
| Psychosis | 1194 | 747 | 223 |
| Pulmonary circulation disease | 590 | 299 | 86 |
| Renal failure, moderate | 1953 | 1214 | 359 |
| Renal failure, severe | 1014 | 849 | 372 |
| Cancer without metastasis, in situ | 1741 | 815 | 178 |
| Cancer without metastasis, malignant | 403 | 154 | 32 |
| Peptic ulcer with bleeding | 404 | 205 | 57 |
| Valvular disease | 2239 | 1064 | 285 |
| Weight loss | 1005 | 533 | 155 |

Figure S5. Relative prevalence of comorbidities by vaccine type. For each vaccine type, the percentage (%) of vaccinated patients that had each comorbidity is shown. For instance, blue represents the percent (%) of Pfizer-vaccinated patients that had each comorbidity. This is obtained by dividing the counts in Table 5 by the number of patients that got the vaccine and multiplying by 100. We see that the relative abundance in comorbidities is similar across vaccine types.

Figure S6. Follow-up duration, following full vaccination, for patients by vaccine-type. The plots show that the minimum number of follow-up days for all vaccine-types is 170 days and the maximum number is 220 days.


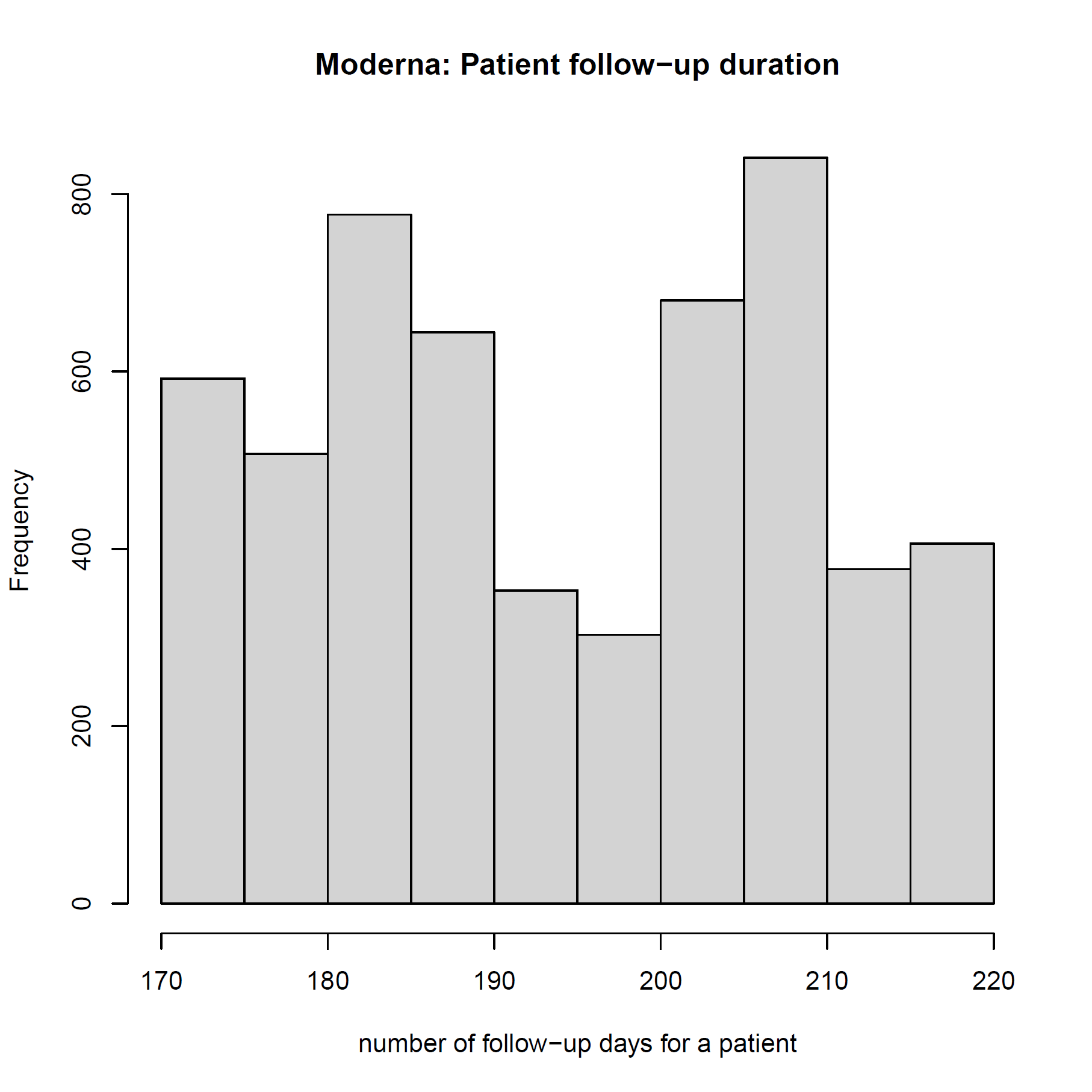

| Variable | Hospitalization Multivariate HR (95% CI)  n=16,725  events=1104 | Mortality Multivariate HR (95% CI)  n=16,725  events=157 |
| --- | --- | --- |
| Vaccine  Pfizer  Moderna  Janssen | 0.42 (0.37--0.50)***  0.41 (0.34--0.5)***  1.0 | 0.45 (0.29--0.69)***  0.40 (0.24--0.67)***  1.0 |
| Age  0-20  20-35  35-50  50-64  64-80  >80 | 0.33 (0.04--2.4)  0.31 (0.16--0.6)***  1.0  2.18 (1.7--2.8)***  3.30 (2.6—4.2)***  4.87 (3.7—6.4)*** | 11.9 (1.07—131.1)*  0.0  1.0  8.67 (2.0—36.2)**  20.5 (5.0—83.7)***  41.7 (10.0—174.0)*** |
| Sex  Female  Male | 1.0  1.25 (1.11--1.4)*** | 1.0  1.14 (0.83--1.56) |

Table S3. Correlates of Hospitalization and Mortality After Breakthrough SARS-CoV-2 Infection, after removing the patients with “prior COVID-19 infection” from the study cohort. Hazard ratios estimated using Cox proportional hazards models. Adjusted hazard ratio (aHR) < 1.0 for the significant correlates (p-values indicated via * or ** or ***), indicates a lower risk of hospitalization or mortality as compared to the baseline population for that covariate, and aHR > 1.0 for the significant correlates indicates a higher risk of hospitalization or mortality.

Figure S7. Number of patients getting hospitalized per month, split by age-group. A much larger proportion of senior individuals were hospitalized as compared to younger individuals.
